# Elastics Selector Gauge as Orthodontics Device Applied to Inter-Maxillary Traction during Malocclusion Correction

**DOI:** 10.3390/jfmk4030063

**Published:** 2019-08-26

**Authors:** Sergio Sambataro, Salvatore Bocchieri, Luigi Bafumi, Luca Fiorillo, Gabriele Cervino, Marco Cicciù

**Affiliations:** 1 Department of Biomedical and Dental Sciences and Morphological and Functional Imaging, Messina University, 98100 Messina ME, Italy; 2 Private practice, 95100 Catania CT, Italy; 3 Multidisciplinary Department of Medical-Surgical and Odontostomatological Specialties, University of Campania “Luigi Vanvitelli”, 80121 Naples, Italy

**Keywords:** elastics, inter-maxillary traction, class II correction, orthodontics, selector gauge

## Abstract

Elastics are the simplest device that can be used during a class correction in orthodontics, and despite the simplicity of a latex band, they are very effective and powerful. The resultant inter-maxillary force affects not only the teeth, but even the mandibular position, and consequently the temporomandibular joints (TMJ). The purpose of our work is to simplify the use of elastics, and to reduce the amount of inventory for orthodontists, because there is a lot of merceology available on the market, and different ways of using the elastics. The use of elastics in clinical practice is based on the force extension values, which are given by the manufacturer for the different sizes of the elastics, generally when they are stretched to three times their lumen size. Various configurations allow for the correction of different malocclusions. We propose a new classification and a new device, the elastic selector gauge, in order to allow clinicians to quickly and easily choose the right elastic in all conditions.

## 1. Introduction

In order to understand the application of inter-maxillary elastics, it would be well to understand things from their beginning. Angle laid the claim to being the first person to use a force from one arch to the other, by the means of a rubber elastic. However, he used it from the lower arch, to an impacted canine on the upper, apparently thinking that the availability of movement of a single tooth was all that was possible.

Since the first cases discussed more than a century ago by Calvin S. Case, and later by Henry A. Baker, who was the clinician who Angle credited with starting the use of elastics in clinical practice—as devices for correcting arch relationships—elastics become a valuable auxiliary of any orthodontic treatment. When combined with good patient cooperation, their use provides the clinician with the ability to correct both antero-posterior and vertical discrepancies.

Prior to that time, orthodontists had attempted to practice “bite-jumping”, with mandibular propulsion as the chief method for correcting Class II malocclusion. When that did not work for Class II correction, they resorted to the extraction of the upper first premolars, for the purpose of gaining space to retract the upper anterior segment. Anterior retraction was frequently done with extra-oral traction, without the thought of molar correction with that modality.

With the demonstration of anchorage from the lower arch to the upper, and with the use of elastics, Angle changed his opinion from extraction to non-extraction, which was to be manifested in his teachings in his career thereafter. He showed that elastics were tied in, and demonstrated that molar correction with inter-maxillary elastics could be achieved in three months [1,2].

The most common type of elastic currently used is the latex type, which is manufactured in the same manner as the India rubber band. A rubber tubing is prepared by a dipping process on a steel mandrel of varying thickness: the more dips, the thicker the tube. The elastic is then sliced from that tubing in varying widths. Manufacturers refer to “light” or “heavy” elastics, depending on the wall thickness of the tubing. Four factors play a part in the tubing quality and characteristics: The size of the lumen of the tubing, the thickness of the wall, the width of the cut, and the properties of the elastic material.

Different factors influence the effects of an elastic on a tooth: such as, the site of application, the distribution through the periodontal ligament, the direction, length, diameter, and contour of the root, the alveolar process, the tooth rotation and health, age, and above all the co-operation of the patient. The force produced by the elastics on a tooth or teeth does not depend only on its magnitude.

Different sizes and force levels are available on the market, but this obliges orthodontists to buy a lot of material; for example, Ormco (Sybron Dental Specialties, Glendora, CA, USA) offers 36 different types of elastic packs, American Orthodontics (Sheboyagan, WI, USA) offers 31 different types, 3M Unitek (Monrovia, CA, USA) offers 30 different types, while Rocky Mountain Orthodontics (Denver, CO, USA) offers 27 different types.

The aim of this study is to give to the profession a simple scientific approach toward selecting the proper elastics for the correction of malocclusions, and to reduce the inventory of orthodontic offices.

## 2. Materials and Methods

The large variety of merceology creates confusion, as it is difficult to select the most efficient elastic in different clinical situations. 

**Figure 1:**
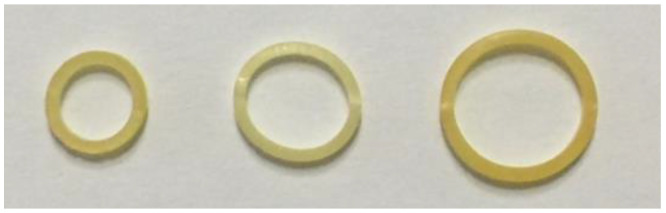
3/16”, 1/4”, and 5/16” 5-oz elastics Energy Pak™ from Rocky Mountain Orthodontics.

We first recommend the adoption of one size of wall thickness, which is small enough to fit under the wing of a bracket, and so for this study we chose the 5 oz elastics, and decided to vary only the length of the elastics in the selection (Figure 1).

Four aspects must be evaluated in order to choose the right elastics for the orthodontic needs of the patients, and they are: 

(1) Distances

In order to have an average of distances in common malocclusions, we studied a sample of N50 class II models (40 cases T1–T2, of which 20 were in full class II, 20 were in neutro-occlusion, and 10 were extraction cases, all in neutro-occlusion). The distances were taken from the distal third of the lower first and second molars, to the mesial third of the upper canines, as the points of application of elastics in a Class II correction. This part is an in vitro study on cast models.

(2) Root mass calculations

**Figure 2:**
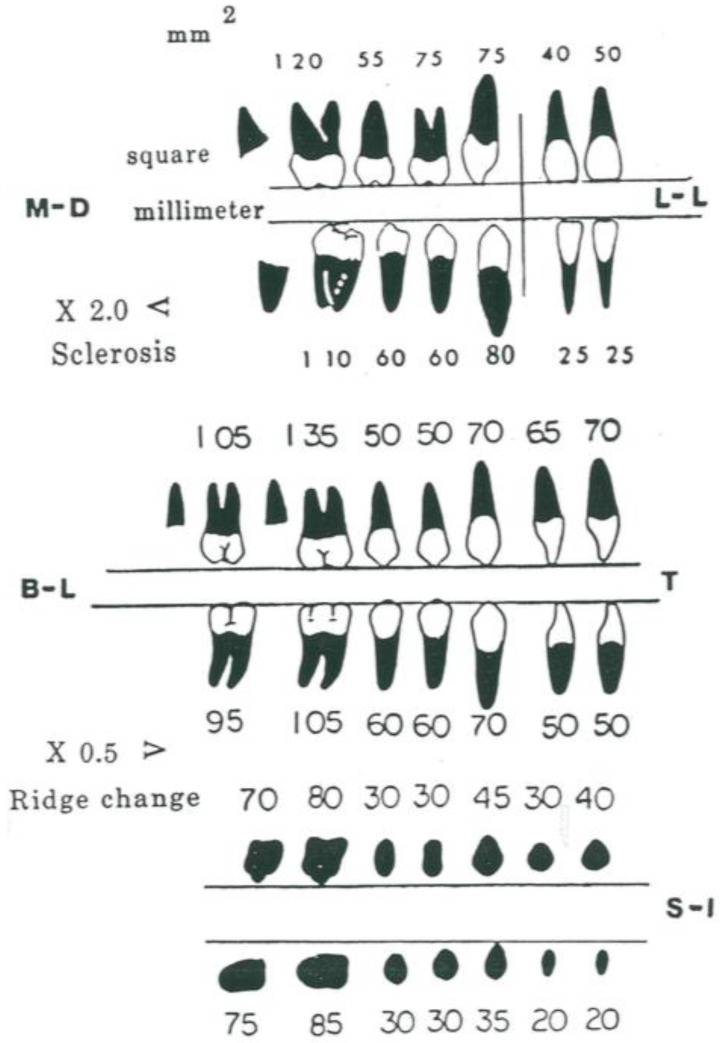
Mean values of root surface presentation on three dimensions of space for permanent teeth: Sagittal, transverse, and vertical. Rotation values are equals to sagittal data.

The amount of force needed for teeth movements can be obtained by calculating from the rating scale for roots (Figure 2). It considers in square millimeters the root surface exposed to the bone, in a direct line of movements. The experiments [3,4,5,6,7,8,9,10,11] led to the conclusion that one gram per square millimeter of root surface is a basic reference for tooth movement. According to these studies, the force to correct the class II malocclusion moving distally for the upper first molar is 120 g.

(3) Vector analysis

An elastic in the right position determines an angle that can be considered with a vector analysis of the force. When vector diagrams are used, the horizontal and vertical components can be calculated. The calculation is made by taking the forces used and estimating the angle of the elastic as it crosses the occlusal plane and then multiplying it by the cosine of the angle for the horizontal, and then the sine of the angle for the vertical pull. As you can see in Figure 2, in the initial condition, the distance of the point of the application of the elastic is longer, whilst the vertical component is shorter, and the horizontal component is greater.

It is for this reason that the elastic is hooked as far distally as possible on the molar, and as far as possible mesially on the canine. During the correction, or in an extraction case, the vertical component will increase and the horizontal component will decrease.

**Figure 3:**
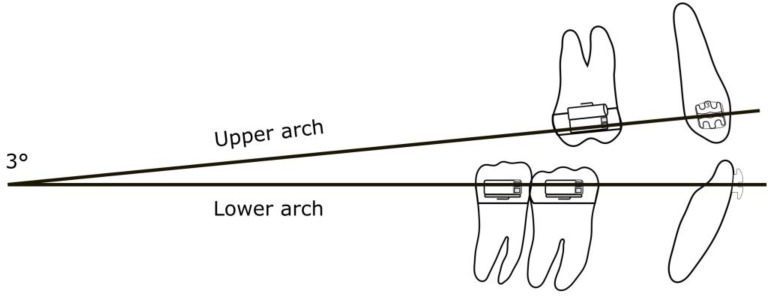
Degrees of mouth opening at rest position.

Different patterns of rest position were studied by Ricketts [12], and the average was 3°, which is important in order to evaluate the reciprocal position of both the maxillary and mandibular arches. In addition, we considered the angle between the elastics and the lower arch wire at different distances (Figure 3).

(4) Decay rate

The decay rate of orthodontic elastics was measured by us in vivo, in three different patients: each wearing two 3/16”, 1/4”, and 5/16” 5-oz elastics (Energy Pak™ from Rocky Mountain Orthodontics, Denver, USA) for a week. Every 24 h, we measured with a dynamometer the force developed from the elastics at different lengths (for the distances that we have studied on casts, see Section 3).

One factor which does seem to be common is the sizing, which has been graduated in 1/16 inch increments. Thus, a graduation exists, such as: 1/8 or 2/16, 3/16, 1/4 or 4/16, 5/16, 3/8 or 6/16, 1/2 or 8/16; which are the usual sizes. For simplification the elastics were labeled #2, #3, #4, #5, #6 etc., referring to each sixteenth of an inch. 

We considered the outer diameter of each of the elastics: The #3 elastic is 6 mm, the #4 elastic is 8 mm, and the 5# elastic is 10 mm. 

For these reasons, we suggest pulling the 5-oz elastics to four times the size of their outer diameter, in order to have a greater range of lengths, with a constant force at the same distance for one week, and with the proper distalization force, considering the following formula:

Fd = Fel − d − Fes

Fd = distalization force

Fel = force of a new elastic

d = 25% decay (Fel × 0.25)

Fdec = decayed force (Fel − d)

Fes = extrusive force (Fdec × sin of angle between arch wires)

In the correction of Class II malocclusion, from a clinical point of view, the only variable that changes in the selection of the elastic is the distance from the molar to the canine. For this reason, it is useful and desirable to have a tool that quickly suggests the correct elastic to use, based on the distance from the molar to the canine.

## 3. Results

We focused our work on analyzing three aspects: The distance, the vector analysis, and the decay of the elastic. We obtained the following results in the aspects investigated in Materials and Methods:

(1) Distance:

We calculated the distances present from the lower molars to the canines in our sample, and we simulated all of the conditions in class II at the beginning of the correction, during the correction, and after the correction, even in the cases where (for orthodontic needs) four premolars were extracted (extraction cases). Identifying the correct distance is the starting point for a correct choice of elastics.

**Figure 4:**
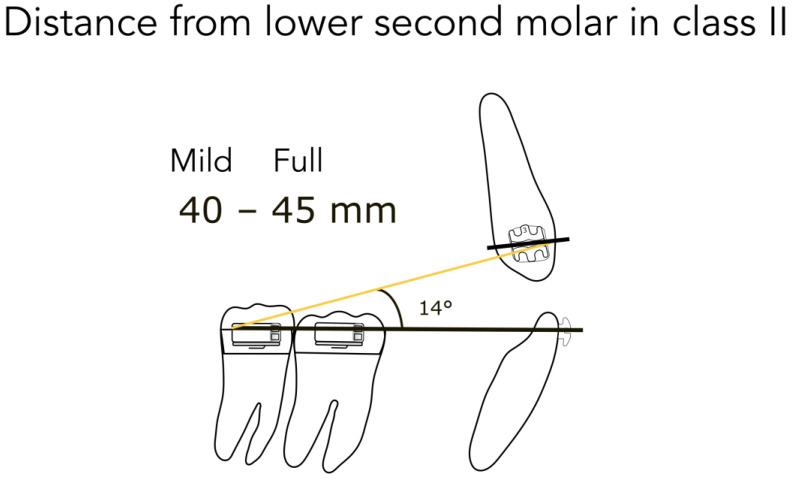
Distance from the lower second molar in Class II.

**Figure 5:**
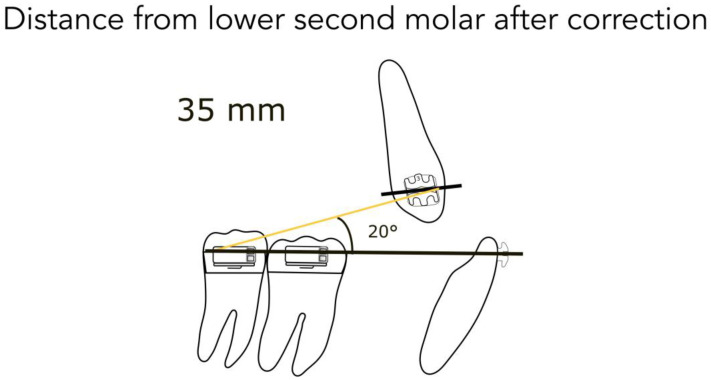
Distance from the lower second molar after correction.

**Figure 6:**
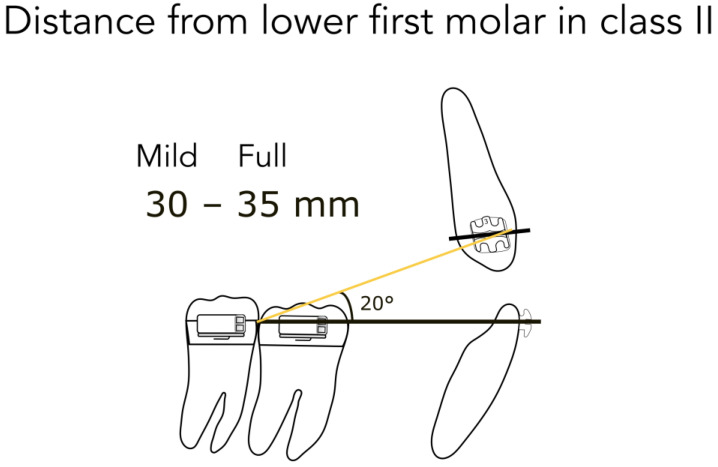
Distance from the lower first molar in Class II.

**Figure 7:**
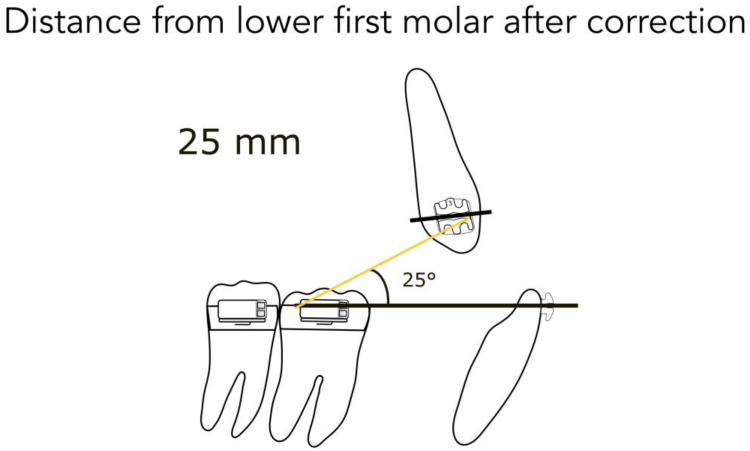
Distance from the lower first molar after correction.

**Figure 8:**
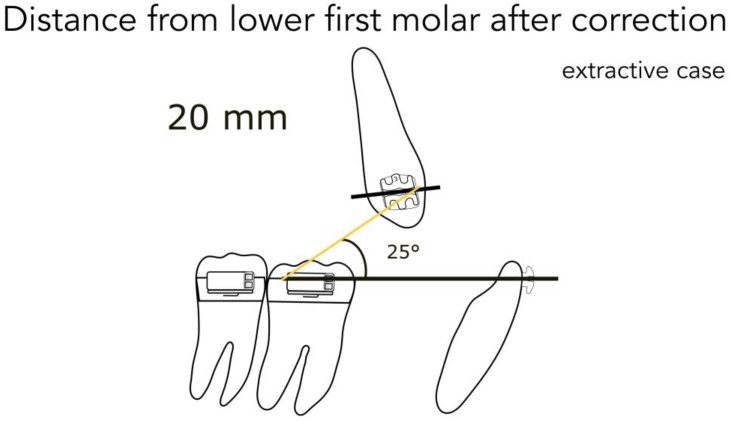
Distance from the lower first molar in the extractive case.

For better understanding, we represent the different kinds of the possible clinical conditions in Figure 4, Figure 5, Figure 6, Figure 7 and Figure 8. These are the averages we found in our in vitro study:

45 mm from the lower second molar in full Class II (Figure 4)
40 mm from the lower second molar in mild Class II (Figure 4)
35 mm from the lower second molar after correction (Figure 5)
35 mm from the lower first molar in full Class II (Figure 6)
30 mm from the lower first molar in mild Class II (Figure 6)
25 mm from the lower first molar after correction (Figure 7)
20 mm from the lower first molar in extractive cases (Figure 8)

(2) Vector analysis:

Consequently, we calculated in our models the angles formed by the lower arch wire, and the elastic applied from the molar to the canine, in order to obtain the vertical component (extrusive), and the horizontal one (distalizing). We found the following results:

for a distance between 40–45 mm, the angle is 14° (second molar) (Figure 4)
for a distance between 30–35 mm, the angle is 20° (first molar) (Figure 6)
for a distance between 20–25 mm, the angle is 25° (extractive cases) (Figure 8)

**Figure 9:**
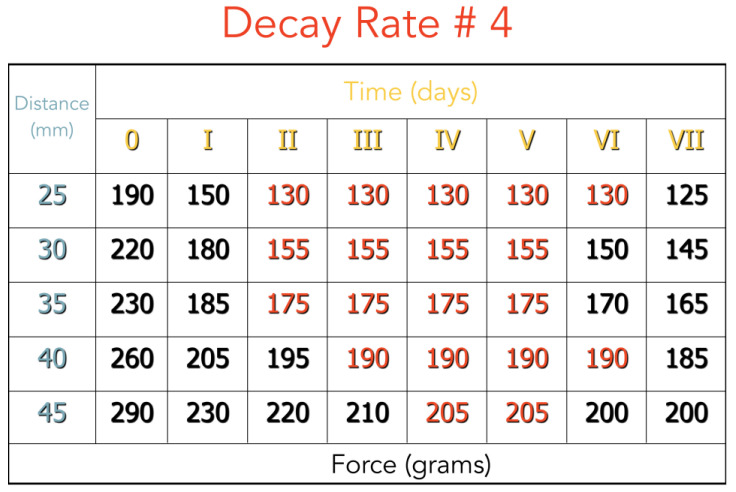
Force delivered by an elastic #4 during a week.

**Figure 10:**
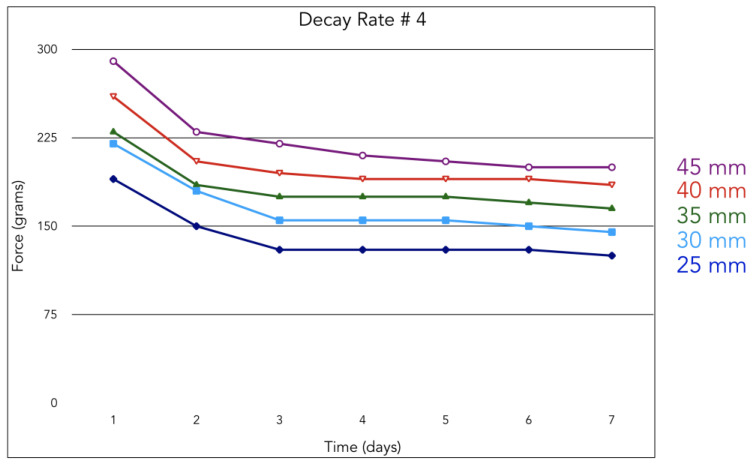
Force delivered by an elastic #4 during a week.

(3) Decay:

We found that the decay rate in one week is about 25%, so the latex elastics can be employed for one whole week maintaining 75% of their pull, which will produce a constant force for a long period, as advocated by many authors [4,7,10]. For this reason, it is advisable to ask to the patients to change their elastics every week (Figure 9 and Figure 10).

The following formulas contain our results for every elastic that we considered:

**#3** Fd = Fel − d − Fes

Fd = 235 − (235 × 0.25) − (176.25 × 0.42)

Fd = 235 – 58.75 – 74.025 = 102.225 gr

Fd = distalization force

Fel = force of a new elastic

d = 25% decay (Fel × 0.25)

Fdec = decayed force (Fel − d)

Fes = extrusive force (Fdec × sin of angle between arch wires 25°) = 24 gr

**#4** Fd = Fel − d − Fes

Fd = 235 − (235 × 0.25) − (176.25 × 0.34)

Fd = 235 − 58.75 − 59.92 = 116.33 gr

Fd = distalization force

Fel = force of a new elastic

d = 25% decay (Fel × 0.25)

Fdec = decayed force (Fel − d)

Fes = extrusive force (Fdec × sin of angle between arch wires 20°) = 32 gr

**#5** Fd = Fel − d − Fes

Fd = 235 − (235 × 0.25) − (176.25 × 0.29)

Fd = 235 − 58.75 − 51.125 = 125.125

Fd = distalization force

Fel = force of a new elastic

d = 25% decay (Fel × 0.25)

Fdec = decayed force (Fel − d)

Fes = extrusive force (Fdec × sin of angle between arch wires 17°) = 40 gr

Studying the parallelogram of force, you can see that the vertical pull over the treatment period will average to about one-third of the oblique pull, while two-thirds of it is horizontal; so, the longer the distance between the ends, where the elastic is attached, the more horizontal the pull becomes. For this reason, it is desirable to apply the elastic from the lower second molar, when it is present, to the upper canine. Thus, you may reduce the vertical component, which results in the extrusion of the upper arch (as an undesirable side effect).

Furthermore, it is also advisable to use the hook extensions incisally on the upper arch, in order to make the pull more horizontal. It is also important to underline that when you stretch the elastics to four times their lumen, the force is the same for the three different types of the elastics that we have used.

## 4. Discussion

Different kinds of malocclusions can be corrected with different approaches and appliances, but in some cases, it is necessary to extract healthy teeth in order to reduce the convexity, and this is extractive therapy [7,8]. In young patients with a dolichofacial pattern, cervical headgear is the best approach to correct the class II malocclusion by orthopedic maxillary alteration, with a superb control of the vertical dimension [13]. In very difficult cases, in which the skeletal discrepancy is too much, or in protocol surgery first [14], the condition could be solved with orthognatic surgery which allows us to treat severe conditions [15,16,17]; but surgery exposes the patients to risks [18,19,20]. Sometimes surgical correction is the only option, but certain orthodontic approaches can reduce the need of maxillo-facial surgery [21,22], and the use of drugs [18,23,24,25] and their side effects [26,27]—considering that the final objective of an orthodontic therapy is the stability of occlusion, and consequently the equilibrium of the kinetic chain of the body [28,29].

Class II correction is one of the most common conditions, especially in the Caucasian race [30,31]. Different strategies of therapy were carried out, but inter-maxillary traction made by elastics is nowadays the simplest strategy, and one of the most efficient, if correctly used [32]. Elastics can be also used for different reasons: To reinforce anchorage in a case where an extraction has been done, to allow the maxillary incisors to move backwards, to correct midline deviation, and to move the lower denture forward. The side effects of Class II elastics should be considered before using them [32].

The common side effects, from the improper use of elastics are: The steepening of the occlusal plane, the extrusion of the lower first molar, the flaring of the lower incisors, and the extrusion of the upper incisors. The first three effects could be avoided by the use of skeletal anchorage, or cortical anchorage. Skeletal anchorage is achieved by using temporary anchorage devices (TADs), which are small screw-like dental implants made of a titanium alloy. As the name implies, they are temporary, as they usually only remain in place during some months of the treatment, and then they are removed. They are placed through the cortical bone in order to become an application point for the elastic traction [33]. Cortical anchorage is a biological method to obtain a valid application site of elastics during Class II correction. When the roots engage the cortical plates, the action will become static and a tooth root becomes a point of resistance, and hence it becomes an anchor for an undetermined period of time. The most efficient tool in order to obtain cortical anchorage is the utility arch. Compact bone not only offers resistance to the tooth movement, but, conversely, it can be used for anchorage, and is recognized and employed to this advantage. This is accomplished by the teeth situated behind the compact elements of bone, so that the pressure of the root is almost in direct contact with the bone, and incapable of easy backward resorption [30].

The extrusion of the upper incisors, with the consequent decreasing of their torque, and the deepening of the bite, produces a gummy smile. This could be avoided by using sectional mechanics, that consist in the cutting of the upper archwire at the level of the canine; thereby excluding the upper incisors from the traction made by the elastics. The two sections obtained could be used to transfer the force applied on the canine directly to the upper molars. Furthermore, the sections could be activated with an intrusion bend that produces 50 g of force, that will prevent the extrusion of the canine as given by the vertical component of the elastic pull.

As we have seen, in order to correct the class II malocclusion, we need 120 g of force to move the upper molar distally. Hence, we chose 5 oz elastics and we stretched the #3, #4, #5, #6 elastics to four times the outer diameter (about five times if we consider the inner diameter), and this produced the same force, which was about 235 g, with the different lengths of elastics. This amount of force must not scare, as by subtracting the correction factors (vector and decay) that we consider in our formula, we have obtained the effective distalization force that we wanted.

Studies of orthodontic elastics have typically used the manufacturer’s recommendations for extending the elastics to three times their lumen, when examining force extension characteristics [34,35,36,37,38]. Some studies used extensions of 20–50 mm, proposing that it was the normal range for clinical use [39,40,41]. In our study, extension distances were obtained from casts of our clinical cases, and we showed that the range is 20–45 mm.

In theory, by evaluating the parallelogram of forces, we find an elastic yield of 75% of force horizontally, and about 25% of the force to be effective in the vertical (with a 20° angle of pull across the occlusal plane). 

In terms of the decay rate, in our measurement (Figure 9) there is a decay of 25% after one day, and then the force remains almost constant for one week, and so the patient could use the same elastic during this period. In this way, a constant force, as advocated by Burstone, is more effective on tooth movements [4].

**Figure 11:**
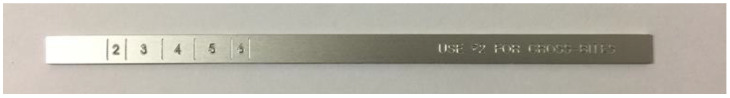
Our “elastic selector gauge”.

By integrating all of the data at our disposal, and by applying the formula used to calculate the force really available for distalization, we created the elastic selector gauge (Figure 11); a device that allows practitioners to easily detect the elastic to be used to treat each kind of malocclusion. It is used by positioning the left end of the instrument at the distal point of the application of the elastic, and then by detecting the number of the elastic required when the mesial point of the application falls between the notches of the instrument. Just three elastics (#3, #4, #5) can correct almost all malocclusions.

## 5. Conclusions

Our classification, in association with the elastic selector gauge, will allow clinicians to make the most effective choice of elastics for class corrections in every condition; without having a big inventory, effort, and waste of time.

## 6. Patents

The results of this work encouraged us to apply for the patent for industrial invention for our device, regularly registered at Italian patent and trademark office on 17/06/2019 with number 102019000009261 entitled: “Elastic selector gauge as orthodontic device: ESGO”.
